# Barriers to cervical screening among older women from hard-to-reach groups: a qualitative study in England

**DOI:** 10.1186/s12905-019-0736-z

**Published:** 2019-02-26

**Authors:** Laura Marlow, Emily McBride, Laura Varnes, Jo Waller

**Affiliations:** Cancer Communication & Screening Group, Research Department of Behavioural Science and Health, University College London, Gower Street, London, WC1E 6BT UK

## Abstract

**Background:**

Cervical screening attendance among 50–64 year-olds is suboptimal. Understanding attitudes to screening and reasons for non-attendance in older women will help to identify the content of interventions for this age group. This study aimed to explore barriers to cervical screening among women aged 50–64 years from hard-to-reach groups whose perspectives are often absent from research on cervical screening but are critical to developing appropriate interventions to increase engagement with the screening offer.

**Methods:**

Qualitative methodology was used. Six focus groups were carried out with women aged 50–64 years from lower socio-economic and ethnic minority backgrounds (*n* = 38). Focus group discussions were recorded, transcribed verbatim and translated where necessary. Data were analysed using the Framework Approach, a type of thematic analysis.

**Results:**

All women had heard of cervical screening, but many felt they had poor knowledge. Women’s reasons for non-attendance were wide-ranging and included discomfort and embarrassment, negative perceptions of health professionals, worry and trust in the results, concern about the procedure, idiosyncratic beliefs, and extreme negative experiences. Some women reported not receiving letters or prompts to be screened.

**Conclusions:**

Information designed specifically for older women should ensure they understand the purpose of screening and its relevance to them. Emphasising changes to the programme that have made the experience less uncomfortable, and improved sample taker awareness of how women feel, may help to allay concerns related to previous negative experiences.

## Background

Globally there are estimated to be around 570,000 new cases of cervical cancer each year and 270,000 deaths from the disease, with the majority of these in low- and middle income countries [1]. In England, around 3000 women each year are diagnosed with cervical cancer, with the peak age for diagnoses currently between 25 and 29 years [2]. The introduction of human papillomavirus (HPV) vaccination is expected to dramatically reduce cervical cancer incidence in vaccinated women and the epidemiology of cervical cancer is projected to change substantially. By 2036–2040 the peak age of a cervical cancer diagnosis in England is expected to shift from late 20s to late 50s [3]. For women in the pre-HPV vaccine cohort (i.e. those born before 1991 in England), participation in cervical screening is their predominant means of preventing cervical cancer and inadequate screening participation at age 50–64 years has been identified as a significant risk factor for cervical cancer for women in their 60s and 70s [4]. However, cervical screening coverage for women aged 50–64 years is considered suboptimal at 77% [5], and has continued to decline over the last 10 years (from 80% in 2007). As a result there has been growing interest in screening policy for women age 50 years and over, with some calling for screening to continue beyond the current stopping age of 64 years [6], and widespread acknowledgement of the need to understand barriers to screening that are specific to older women [6, 7].

A recent review aimed to synthesise the evidence relating to psycho-social influences on attendance at cervical screening among older women [7]. Drawing on data from 22 studies, knowledge of cervical screening among women in the 50–64 age group was generally poor, and barriers to screening encompassed a range of different factors including embarrassment, procedural concerns (e.g. about male screeners and discomfort/pain), past experience, low perceived need due to feeling well and not having symptoms, general distrust of the medical profession and practical barriers (e.g. appointment scheduling). However, since most of the studies included women across the whole screening age range, it remains unclear to what extent these barriers were specific to older women. Recent work that focuses on older women has suggested they may be more likely to find the test uncomfortable due to physiological changes post-menopause (e.g. vaginal atrophy) [8, 9]. And may also see themselves as being at lower risk of cervical cancer if they have had a single sexual partner for a long time or are no longer sexually active [10]. A study that divided non-participants at screening into different groups, suggested that older women were more likley to have decided not to attend screening in the future [11].

Women who do not attend for cervical screening are also more likely to be from lower socio-economic status (SES) and ethnic minority backgrounds [11, 12]. This has been consistently shown across the screening age range, but can also be seen more specifically within the 50–64 year age group [13]. Women in these population sub-groups are considered ‘hard-to-reach’ for the purposes of research and their views can often be underrepresented. Several studies have explored attitudes to screening among ethnic minority women [14–16], but none of these have focused on older age groups.

The objective of the current study was to draw together the need to understand the views of women aged 50–64 years and concerns about ethnic and socio-economic inequalities in screening. We aimed to explore barriers to attendance at screening among older women from ethnic minority and lower SES backgrounds. Given the lack of research with the particular population we chose to carry out an exploratory qualitative study to identify the main themes that arise from women’s open discussion of this topic.

## Methods

### Design

This study used a qualitative design. Data were collected using focus groups.

### Participants

Participants were women aged 50–64 years. Women aged 50–64 years are classified as being in the ‘older’ of the two screening age cohorts in England and are invited every 5-years (women aged 25–49 years are invited more frequently). We particularly wanted to recruit women from ethnic minority and lower SES backgrounds who are often under-represented in this type of research. For the purpose of this research ethnic minority women were considered to be women who classified their ethnic background as anything other than white British (this is in line with how ethnic minority is classified using English census data) [17]. We initially planned to recruit eight groups of women; four groups with women from ethnic minority and four with low SES backgrounds. This number took into account the scope of the study, the nature of the topic and recommendations about feasible sample size [18].

Four focus groups with ethnic minority women were organised through community groups. Community groups indicated their interest in helping with the research after seeing an advert describing the project that was posted on an online community group network page. Community leaders were asked to recruit women and were offered a financial donation for helping to do this. For the focus groups with women from lower socio-economic backgrounds women who were on a pre-existing panel maintained by a market research panel and met the required criteria were contacted and invited to participate in a focus group. The required criteria were being aged 50–64 years and from social grade C (lower middle class or skilled working class); D (working class) or E (non-working). Ultimately we were only able to recruit two groups of women from low SES backgrounds.

### Procedure

Data collection took place between February and November 2017. Focus groups were moderated by EM and were held at University College London (UCL) or at a community group venue familiar to the women. Upon arrival participants were given a study information sheet to read and a consent form to sign and completed a short questionnaire assessing their age, marital status, work status, education, religion, ethnicity and cervical screening history. To assess cervical screening history women were told “Women aged 50-64 years are offered cervical screening (also known as smear or Pap tests) every 5 years” and then asked three questions: 1) Have you ever had a smear test? (yes/no/don’t know), 2) How long ago was your last test? (less than 5 years/more than 5 years/Don’t know). 3) Next time you are invited for a test do you think you will go for it? (yes/no/don’t know).

A topic guide was developed for the focus groups exploring experiences of cervical screening and attitudes to attending in the future. A photograph was also used to help women understand what the screening procedure involved. Open-ended questions were used to encourage in-depth responses. The study was approved by the UCL Research Ethics Committee (ref: 0496/014).

During recruitment through community centres, the need for interpreters was discussed with the community leader. When it was anticipated that an interpreter was necessary the community leader identified someone to fulfil this role (usually a member of staff from the centre). Interpreters also helped women in the group to complete the paperwork. Women were offered a cash incentive of £25 for taking part in a focus group (to cover time and travel expenses). All women signed a consent form before participating in the study.

### Analyses

Recordings were transcribed verbatim and, when in another language, translated into English (by an external agency). Data were organised using the Framework approach [19], which facilitates data management and allows for thematic analysis of the data to be carried out. Thematic analysis is a method for identifying, analysing and reporting themes identified within the data [20, 21]. It is a well-structured approach which allows examination of perspectives across different participants. A theme is not necessarily dependent on quantifiable measures but rather on whether it captures something important in relation to the overall research question and while researchers can highlight similarities and differences between participants the intention is not to establish evidence of statistical difference. Two researchers (EM and LV) initially read and re-read the first two transcripts to familiarise themselves with the data. They then independently coded the transcripts line by line and shared codes to check consistency. The codes were then used to develop a coding framework which was used to code the next four transcripts (by LV). Additional codes were added to the coding framework where necessary. LM also read the manuscripts and cross-checked these with the final coding frame. A matrix was created to collate the data (in Excel). Interpretation of the data involved inspection by group and theme. Four women were slightly outside the screening age range but since their discussions are within a group we have included them. We have provided a detailed description of the themes that were identified in the data, with example quotes. Frequency counts are not presented.

## Results

### Sample characteristics

A total of 38 women took part in the study, participating in one of six focus groups. Four of the groups were in English or mostly English, one group was in Sylheti and one group was in Arabic/Somali. Focus groups lasted between 50 and 94 minutes. The mean age was 56.7 years (range 49–67) and about half of the women were married or cohabiting (*n* = 18). Half were educated to secondary school level (*n* = 19) and the majority were not working (*n* = 29). Women were from a range of ethnic backgrounds including Bangladeshi, Pakistani, African, Caribbean and White British. Most women had been for cervical screening before (*n* = 33) but around half were currently overdue for their screening test (n = 19) and just over half were not planning on or not sure about going for screening in the future. Table 1 shows the socio-demographic characteristics and the self-reported screening histories of the women who took part.

**Table 1:** Participants’ demographic characteristics

	Group 1 (*n* = 4)	Group 2 (*n* = 4)	Group 3 (*n* = 6)	Group 4 (*n* = 6)	Group 5 (*n* = 9)	Group 6 (*n* = 9)^a^
Age (mean, range)	56 (51–59)	59 (54–65)	55 (49–60)	51 (50–54)	57 (50–67)	61 (50–67)
Marital status						
Single	2	3	0	0	5	0
Married/cohabiting	1	1	4	3	2	7
Divorced separated/widowed	1	0	2	3	2	1
Employment^b^						
Employed full-time	1	0	0	0	0	0
Employed part-time	2	1	1	1	1	0
Unemployed	0	0	5	3	4	4
Full-time homemaker	1	0	0	1	1	0
Retired	0	2	0	0	0	3
Student	0	1	0	0	0	0
Too ill to work	0	0	0	1	3	0
Education^b^						
No formal qualifications	0	1	0	0	2	3
Secondary school level	3	2	5	4	6	0
University degree	1	1	1	1	0	1
Other	0	0	0	1	1	2
Ethnicity^b^						
White British	3	2	1	0	0	0
Black African	0	1	0	4	7	0
Black Caribbean	1	1	0	0	0	0
White & Black African	0	0	0	2	0	0
Pakistani	0	0	2	0	2	0
Bangladeshi	0	0	1	0	0	7
Have you ever had cervical screening?						
Yes	4	4	5	6	7	7
No/Don’t know	0	0	1	0	2	1
How long ago was your last test?						
Less than 5 years ago	1	1	4	3	5	4
More than 5 years ago/don’t know	3	3	2	3	4	4
Next time you are invited for a test do you think you will go for it?						
Yes	3	0	1	1	4	7
No/Don’t know	1	4	5	5	5	1

^a^ *One participant did not complete the questionnaire*

^b^ *Total n < 37 because some questions were unanswered*

### Thematic structure

The three major themes reported here were driven by the discussion guide and include: 1) knowledge of cervical cancer risk factors, 2) knowledge of cervical screening and 3) barriers to attending screening. Within each of these a number of key sub-themes were identified. These sub-themes are discussed below with supporting quotes (additional supporting quotes are included in supplementary tables). Participant identifiers and focus group numbers are reported, M = moderator and I = interpreter). Groups 1 and 2 were predominantly with women identified from lower SES white backgrounds and groups 3–6 were predominantly with ethnic minority women.

#### Knowledge of cervical cancer risk factors

In general, many of the women felt they had poor knowledge of cervical cancer. Many women who were from ethnic minority backgrounds highlighted that there was a widespread lack of awareness about cervical cancer in their countries of birth, and that it is not usually screened for.

##### Sexual behaviour

In four of the six focus groups some women mentioned sexual intercourse as a risk factor for cervical cancer. More specifically, it was suggested that having multiple sexual partners, having ‘too much sex’, or having sex at a young age could increase the risk of cervical cancer;

*“If a lady have sexual relationship with different men in short period of time that could cause”* (P1, Group 4).

Some women from ethnic minority groups appeared to abstain from the discussion around the role of sexual behaviours in cervical cancer development and in Group 6 (where all women were from Bangladeshi backgrounds), sexual behaviour was not mentioned at all.

##### Age

Women had varying views about whether age increased or decreased risk of cervical cancer and some provided possible mechanisms for why this might be. For example, one woman who thought cervical cancer risk decreased with age suggested this was because the cervix erodes:

*“You lose, what is it, the ovaries … your actually cervix etcetera actually erodes away a bit … but I presume you have less chance because you’d have presumably less cervix, or whatever”* (P2, Group 1).

Another woman who believed that age increased the risk of cervical cancer thought this was due to hormonal changes:

*“I do believe our chances go up now of getting it. Just because the hormonal change, the oestrogen and that and so you, they might wear away cells at a different rate”* (P3, Group 1).

##### Other causal beliefs

Other suggestions of factors that increase the risk of cervical cancer were varied and dispersed between groups. They included smoking, alcohol, ethnicity, obesity, having children, fibroids, lacking vitamins, breastfeeding, chemicals in soap, vaginal dryness, hormonal changes, negative emotions, diabetes, and urine infection. One woman mentioned ‘papilloma virus’. Four of six focus groups discussed how family history of cervical cancer could predispose or increase the risk of cervical cancer; *“I think it’s something that’s passed down … So, if you have someone in the family you’re more likely to develop it.”* (P4, Group 1).

A small number of women from ethnic minority backgrounds believed that using condoms could increase the risk of developing cervical cancer. This was based on beliefs that, in their countries, condoms could cause diseases; “*… there’s a time where you did HIV/AIDS campaigns in Zimbabwe. There was that some kind of say that condoms were bringing a lot of diseases to people.”* (P7, Group 5). However, one women disagreed and felt that condoms were safer in the UK because they were tested to ensure they met a certain standard. Some women, particularly those from ethnic minority backgrounds, described fatalistic beliefs about cervical cancer, feeling that a cervical cancer diagnosis would be beyond their control; *“I just think anyone can get it. Sort of like whether you’re really healthy or whatever, you still, there’s a slight risk that you could have it. If you’re gonna get it you’re gonna get it”* (P1, Group 3). Other women believed that there is a level of control over developing cervical cancer because they have control over whether they attend cervical screening and whether they have multiple partners or unprotected sex (mentioned in three groups).

#### Knowledge of cervical screening

Whilst all women who participated in the focus groups had heard of cervical cancer screening, there was evidence of poor knowledge and understanding in some of the groups.

##### The purpose of screening

A number of women held basic knowledge on cervical screening and were aware that it was carried out to detect abnormal or pre-cancerous cells in the cervix, helping to detect cervical cancer in its early stages and prevent it from spreading,

*“I don’t know very much about [cervical screening]. I know is, all I know is that you can get pre-cancerous cells and they can be found from smear tests. And that’s before they become cancerous, and they can be treated really quickly.”* (P1, Group 1).

A number of women from ethnic minority backgrounds were not able to specify the purpose of cervical screening. Some women suggested that it was used to investigate ‘how cells are behaving’ or to find out whether women are ‘healthy down there.’

##### Screening eligibility

Most women thought that cervical screening was offered every 3–5 years, however there was uncertainty in their answers. In one of the focus groups with women from ethnic minority backgrounds there was uncertainty about what age cervical screening is initially offered. Women from this group suggested that cervical screening is performed after a woman gets married, ranging from 20 to 40 years of age;

Group 6 excerpt:

P0: *“After marriage it is done for every girl. It is done according to age. It’s also done after 40. I know that this test is also done after becoming 20 years old. Our doctors told us when to perform this test.”*

I: *“She was saying after marriage. But most of them are saying 40, 30, 35 like that.”*

##### Reasons for poor knowledge

In one of the focus groups with women from ethnic minority backgrounds stigma and fatalism were discussed as contributing to poor knowledge about cervical cancer and screening. One woman in this group discussed a common ‘myth’ that cervical cancer was a disease people got as a result of having ‘done bad things’ and this meant it was usually not discussed,

Group 3 excerpt:

P6: *“The stigma, why I think, because people don’t have much information about this problem, the most perception about cervical cancer within the community is that this disease is someone suffered from this disease having had bad lifestyle or bad things. So that’s why.”*

P1: *It’s a shame isn’t it?*

P6: *Yeah, it’s such a shame. It’s definitely a shame. So they think this problem comes because of that bad things. They don’t think that this is also a problem, er it’s a medical condition, it can happen to anyone.*

#### Barriers to attending cervical screening

Women’s discussions about barriers to cervical screening included seven sub-themes: i) value of screening, ii) risks of the procedure, iii) discomfort and embarrassment, iv) extreme negative experiences, v) health professionals, vi) concern about results, and vii) opportunities.

##### Value of screening

A few women believed that they would know if there was something wrong and therefore screening was not necessary. Others believed that cervical cancer was not treatable and so they would prefer not to know if they had it; *“You have feeling that if it’s a cancer it’s not treatable, so I leave it, I don’t want to know”* (P3, Group 3). Poor knowledge and misconceptions of cervical cancer, particularly in the ethnic minority focus groups, were highlighted as factors influencing perceptions of the value of screening. For some, these misperceptions influenced perceptions of risk with the belief that cervical cancer runs in families meaning they did not have to worry.

##### Risks of the procedure

Some participants were concerned that there could be a chance of cross-contamination through use of equipment that had not been sterilised:

*“Something else has bothered me as well, I think that … I don’t know, like you call them instruments or whatever it is they use. I’m not always so sure they are as clean as what they say they are”* (P4, Group 2).

One woman from an ethnic minority background was concerned that the health care professional might put cancer inside her during the screening process, or that the test itself might cause cervical cancer; *“If you go there, they’ll plant cancer in you.”* (P3, Group 5).

##### Discomfort and embarrassment

The majority of women reported that they found cervical cancer screening ‘uncomfortable’, ‘embarrassing’, and ‘painful’. This was specifically related to pain during insertion of the speculum and the feeling of embarrassment due to the sensitive nature of the screening process; *“It’s a very sensitive area, the private area, for every woman”* (P6, Group 6). In general, most felt screening had always been uncomfortable and this had not changed with age, but some women reported that they had experienced more pain during screening since the menopause which they attributed to vaginal dryness. Although still describing the process as uncomfortable, a few women found the process acceptable due to the short time it takes to complete. These women felt the negatives aspects of screening were worth the health benefits: *“What’s a few minutes of discomfort for our health, yeah”* (P1, Group 1).

##### Extreme negative experiences

In five of six focus groups, women mentioned that a previous bad or painful experience of cervical screening had ‘scared’ them and put them off attending again in the future:

*“The pain was really bad and we had to stop it”* (P1, Group 2).

*“I suppose from that onset of having the bad experience I haven’t liked it. And I’ve sort of like, if I can I’ve always avoided it”* (P2, Group 1).

In one focus group, some women from ethnic minority backgrounds discussed how the experience of cervical cancer screening brought back memories of female genital mutation (FGM) and childbirth. These memories included the emotional and physical trauma of FGM (including pain and physical injury), and the environmental context which was viewed as similar to childbirth and the positioning required for screening. These negative experiences beyond the screening context influenced their anticipated likelihood of attending in the future:

*“For me it reminds me the doctor, they put me like this and they touch me and they grab my legs and I start screaming and seeing blood. Everything is coming back. I wept when they sent me letter to go to do this. I hate it. I feel like I want to hit somebody, I want to scream or because of all the things I’ll never ever forget … that feeling is there”* (P2, Group 4).

##### Health professionals

Women from lower socio-economic status backgrounds felt that the ‘clinical environment’ and health professional’s character and level of expertise could influence their experience of screening. Many described the health professional performing the cervical screen as the ‘most important thing.’

Group 1 excerpt:

P4: *In terms of, erm, the experience of it overall is not very pleasant. But also as, and, well I feel like the environment that it’s set in is, is not nice either. I mean, I’m not, you know, expecting like flowers and, and beautiful colours and everything else but it just all feels very* P2: *Clinical.*

P1: *Clinical.*

P4: “*Yeah. And depressing as well. It’s just like, erm, it’s, it’s a routine for the person doing it, so there’s no … do you know what I mean, like, erm, interaction as such. I don’t know.”*

The health professional’s ‘bedside manner’, level of empathy, and experience and ability to perform cervical screening without causing undue pain were important factors in women’s memories of previous screening appointments and their anticipated experience of future ones: *“Mine was absolutely horrible and, erm, that’s why I won’t go again. It was … the first time I ever had it done it was okay but the second time I went, I don’t know quite what happened and I thought I was gonna die from the pain from this woman and then … and I did cry. I mean, it hurt that much. And she shouted at me and called me a baby, err, which was just dreadful.”* (P2, Group 2).

An ability to develop a rapport, and ensuring women felt ‘at ease’ were considered important. Women also felt they might benefit if the health professional informed them that discomfort is normal, therefore normalising their experience:

Group 2 excerpt:

P2: *“And this empa- empathy is what you need and, I mean, I’d far rather somebody was honest and said, ‘This will hurt,’ or, ‘This could very well hurt,’ because it doesn’t for everybody, than, ‘It’s not gonna hurt and, you know, sort of get a move on, get real,’ that kind of attitude”.*

P4: “*No, no, definitely, I agree with this lady. I think that if, if they even said like, ‘It may hurt, just to let you know in advance,’ and therefore I think you would have that in your mind then at that point, wouldn’t you, and you’d be like, okay, well you’d be prepared for it I think. And if it doesn’t then fine but if it does then you know”.*

##### Concern about results

A few women from ethnic minority backgrounds also expressed worry about getting the results. One woman raised concerns of lack of information in the letter if you are re-called for another test, noting that this can be anxiety provoking. Trust in cervical screening results was discussed briefly in a few of the groups. A number of women mentioned that due to knowledge of false positives and false negatives they would have difficulty trusting the results:

*“It’s just such a horrible thing to have done. And as I said, I’m not, you know, entirely confident in the result. It just seems to be … Well, you know I could go and have this, and I mean, if I did get sort of an, a positive result, I could start a whole thing off and find there’s nothing there anyway.”* (P2, Group 1).

##### Opportunities

Another common barrier to screening attendance was no longer receiving an invitation letter. This was mentioned in five of six focus groups. In the lower socioeconomic groups women reported that lack of insistence by health professionals and lack of follow-up following non-attendance of cervical screening influenced their attendance of screening appointments:

*“Nobody told me to go [to screening], anymore. The GP didn’t write to me, erm, no nurse told me. I have regular doctors’ visits every once a month”* (P2, Group 3).

One woman reported that she has been informed by her general practitioner (GP) that she no longer needed to attend screening appointments:

*“I said, ‘You know, I find this whole process really uncomfortable’. I can’t remember how old I was at the time because I can’t actually remember how long ago it was. ‘Do I need to keep coming for these? Erm, I’m not in a regular … relationship and I’ve never been called back from a, a previous smear test.’ He said, ‘Err, on that basis, I, I think you can quite safely not have any more, it’s, it’s unnecessary for you to have any more.”* (P1, Group 2).

Women in these groups also highlighted other barriers including difficulty in finding time to attend, and inconvenient location and appointment times making attendance problematic. Furthermore, one woman from a lower socioeconomic status background reported actively avoiding health professionals who might suggest attending screening and pushing thoughts about it to the back of her mind.

## Discussion

This work explored beliefs about cervical cancer and screening, as well as barriers to attending screening among older women (aged 50–64 years) from lower socio-economic and ethnic minority backgrounds. Few studies have focused on understanding psycho-social influences on cervical screening uptake in this older age cohort [7], despite a clear need for this [6]. This work is one of the first studies to explore themes that arise in discussion about cervical cancer among older women from low SES and ethnic minority backgrounds. By targeting our recruitment efforts to these ‘hard-to-reach’ groups, we were able to gain insight into the perspectives of women who are rarely included in research of this kind. The themes identified point to issues that might be explored in future quantitative studies and could be addressed in interventions to improve attendance in this group.

The women in our study generally had poor knowledge of cervical cancer risk factors. This is consistent with a number of quantitative studies that show cervical cancer risk factor knowledge is poor, particularly among women from more deprived backgrounds [22]. While sexual behaviour was explicitly mentioned as a risk factor for cervical cancer in some of the groups, one of the groups with women from ethnic minority backgrounds did not mention this at all. Despite being the primary casual factor for cervical cancer, HPV was rarely discussed. The introduction of HPV testing and the information that accompanies this may result in improved knowledge but also has the potential to cause confusion (at least initially) among women to whom it is unfamiliar.

Improving knowledge of cervical cancer risk factors, focusing on the relevance of these factors to women in the older age, and de-bunking beliefs that undermine perceived personal relevance of screening (e.g. that screening is for recently married women) would help women to make more accurate judgments about their risk of cervical cancer and the benefits of being screened.

Previous studies have suggested that some ethnic minority women are not familiar with the term cervical screening or smear/Pap [15], so we used a photograph to clarify what the screening procedure involved. Following this all the women indicated that they were aware of cervical screening, but knowledge about the purpose of screening was basic for some of the women, and this was especially the case in the focus groups with ethnic minority women. This is consistent with a number of previous studies which show poor understanding of cervical screening among women from ethnic minority backgrounds [15, 23, 24]. Culturally-specific beliefs including fatalistic beliefs about getting cervical cancer were also touched on by some ethnic minority women, although these were not as widespread as other studies have suggested. Many of the women were uncertain about eligibility and age intervals for cervical screening among older women and none mentioned the approaching eligibility ceiling (i.e. that screening would no longer be offered to them over the age of 64 years).

Women discussed a wide range of barriers to screening, mostly similar to those described in other studies [7]. This suggests that interventions need to address multiple aspects of the screening process to make it more acceptable to women in the older age group. These themes ranged from concerns about the procedure that may be easy to address with reassuring messages (e.g. that the equipment is sterile and disposable, and a positive result rarely means cancer), to extreme negative experiences of cervical screening specifically, or other experiences (e.g. childbirth/FGM), which may be more of a challenge. Interestingly, practical barriers to screening did not appear to be a prominent barrier to screening in any of the focus groups. The focus on experiences over practical barriers supports findings from previous qualitative work which found that older women were less likely to describe difficulties making appointments or competing demands on their time, but more likely to describe low risk, worry and concerns about embarrassment and pain [10].

In a recent review of qualitative work, two broad themes were described ‘Should I go for screening?’ (which focused on perceived risk and value of screening) and ‘screening is a big deal’ (physically and emotionally) [25]. In the current study there was very little discussion about the perceived relevance of screening and none of the women who had decided not to attend in future described making what could be ‘informed decisions’ (i.e. weighing up the risks and benefits). In most cases the decision not to go for screening in the future was based on previous bad experiences (i.e. seeing screening as a ‘big deal’). Since some of these experiences may have been many years ago, informing women about changes to the screening process which are designed to make it less uncomfortable (e.g. plastic speculums, different size speculums), as well as new guidelines for ‘cervical sample takers’, ensuring they have a better understanding of the individual’s perspective [26], might help to address some of these concerns. HPV self-sampling or clinician-collected HPV sampling (without a speculum) may be key approaches for overcoming dislike of the screening procedure among older women. A recent study in Belgium showed that around 20% of women > 50 years old who did not participate in screening returned an HPV self-sampling kit when it was sent to them [27]. In addition, discussion with a health professional may have more impact for lower SES and ethnic minority women, especially where understanding of the purpose of cervical screening is poor. The detailed explanation of screening could also ease concerns about the procedure.

In general, the broader themes were similar for ethnic minority and lower SES women, although exposure to particular experiences and community beliefs influenced the origin of these. For example, negative experiences were described across all groups but for ethnic minority women this was (in some cases) influenced by experience of FGM. Worry about test results was mentioned by lower SES and ethnic minority women, but for some ethnic minority women (from African backgrounds) this stemmed from feelings about being tested for HIV/AIDS. Training community leaders to discuss these culturally-specific concerns with women may be helpful.

### Limitations

Women were recruited from lower socio-economic and ethnic minority backgrounds, both traditionally hard-to-read groups, often underrepresented in research. Low-SES and ethnic minorities often overlap and this was the case in the current study. While two of the focus groups were recruited as low SES groups, some of the women in these groups were also from ethnic minority backgrounds and most of the ethnic minority women could also be classified as low SES. Since we did not focus on one specific ethnic group, we cannot see in-depth insights into one specific ethnicity. In addition we did not collect data on length of time in England or on religion and so could not explore the role these variables might play.

Qualitative work is not intended to be representative of the population, rather it allows the identification of a range of views. There is likely to be participation bias, with women who are more open to discussing their experiences more likely to agree to participant. We did not assess women’s history of cervical screening results so do not know the role this might play.

Since this is a small qualitative study we cannot describe how common each of these themes are likely to be among older hard to reach women more generally. Future research may help to quantify the prominence of the themes highlighted here. The themes that women discussed here could be used to design a questionnaire and collect quantitative data from the target group. Collecting quantitative data would also allow for exploration of potentially confounding variables.

## Conclusions

Information designed specifically for older women should ensure they understand the purpose of screening and its relevance to them. In particular, the benefits of screening between 50 and 64 years for reducing future risk of cervical cancer should be communicated. Older women from lower SES and ethnic minority backgrounds describe decisions not to attend screening often on the basis of bad experiences and worry about the test procedure and results. Emphasising changes to the programme that have made the experience less uncomfortable, and improved sample taker awareness of how women feel, may help to allay these concerns.

## Abbreviations

FGM: Female genital mutilation
GP: General practitioner
HPV: Human papillomavirus
SES: Socio-economic status
UCL: University College London
